# Effects of a 4-week high-intensity interval training on pacing during 5-km running trial

**DOI:** 10.1590/1414-431X20176335

**Published:** 2017-10-19

**Authors:** R. Silva, M. Damasceno, R. Cruz, M.D. Silva-Cavalcante, A.E. Lima-Silva, D.J. Bishop, R. Bertuzzi

**Affiliations:** 1 Grupo de Estudos do Desempenho Aeróbio (GEADE-USP), Departamento de Esportes Escola de Educação Física e Esportes, Universidade de São Paulo, São Paulo, Brasil; 2 Grupo de Pesquisa em Ciência do Esporte, Faculdade de Nutrição, Universidade Federal de Pernambuco, Pernambuco, Brasil; 3 Grupo de Pesquisa em Desempenho Humano, Universidade Tecnológica Federal do Paraná, Paraná, Brasil; 4 Institute of Sport, Exercise and Active Living, Victoria University, Melbourne, Victoria, Australia; 5 School of Medical and Health Sciences, Edith Cowan University, Perth, Australia

**Keywords:** Rating of perceived exertion, Running economy, Peak treadmill speed, Maximal oxygen uptake

## Abstract

This study analyzed the influence of a 4-week high-intensity interval training on the pacing strategy adopted by runners during a 5-km running trial. Sixteen male recreational long-distance runners were randomly assigned to a control group (CON, n=8) or a high-intensity interval training group (HIIT, n=8). The HIIT group performed high-intensity interval-training twice per week, while the CON group maintained their regular training program. Before and after the training period, the runners performed an incremental exercise test to exhaustion to measure the onset of blood lactate accumulation, maximal oxygen uptake (VO_2_max), and peak treadmill speed (PTS). A submaximal constant-speed test to measure the running economy (RE) and a 5-km running trial on an outdoor track to establish pacing strategy and performance were also done. During the 5-km running trial, the rating of perceived exertion (RPE) and time to cover the 5-km trial (T5) were registered. After the training period, there were significant improvements in the HIIT group of ∼7 and 5% for RE (P*=*0.012) and PTS (P*=*0.019), respectively. There was no significant difference between the groups for VO_2_max (P*=*0.495) or onset of blood lactate accumulation (P*=*0.101). No difference was found in the parameters measured during the 5-km trial before the training period between HIIT and CON (P*>*0.05). These findings suggest that 4 weeks of HIIT can improve some traditional physiological variables related to endurance performance (RE and PTS), but it does not alter the perception of effort, pacing strategy, or overall performance during a 5-km running trial.

## Introduction

It has been widely recognized that during recreational and official athletic events the running intensity is always self-selected by athletes (1–3). The manner by which runners self-select their running speed during a given competition has been defined as pacing strategy (4). Specifically, during a 5-km running race, athletes usually adopt a pacing strategy characterized by a fast start (first 400 m), followed by a period of slower speed during the middle part (400–4600 m), and a significant increase in running speed during the last part (final 400 m) of the race (2). These variations in running speed seem to occur to optimize the use of the available energy resources (5). Based on the linear increase in the rating of perceived exertion (RPE) during time-trials, some studies have suggested that this triphasic pacing strategy profile (so-called “U-shaped”) could reflect a centrally-regulated control system (1,2). It is believed that athletes might consciously monitor their RPE based on internal (physiological) signals and change their running speed in order to prevent a premature exercise termination (6,7).

Previous studies have observed a significant relationship between traditional physiological predictors of endurance performance and running pacing strategy (7–9). Lima-Silva et al. (9) reported that runners with a higher running economy (RE), peak treadmill speed (PTS), and a faster speed corresponding to onset of blood lactate accumulation (OBLA) presented a more aggressive U*-*shaped speed curve during a 10-km running race compared with their counterparts. In addition, high-performance athletes ran the first 1200 m of a 10-km race at a speed faster than the average speed of the entire race and above their PTS, while a low-performance group started the race with a less aggressive pacing strategy and slightly below the OBLA speed (9). These results suggest that athletes with higher PTS, OBLA, and RE may be able to improve their performance by increasing mainly their speed during the first part of a running race. According to the theory suggesting that the exercise intensity is regulated by the central nervous system (CNS), an improvement in these physiological variables could enable athletes to begin the race with a higher starting speed without provoking critical changes in homeostasis that otherwise could lead to premature fatigue.

It is also well recognized that physical training produces a number of changes in the metabolic function in different physiological systems (10,11). Specifically, the addition of a short-term, high-intensity interval training (HIIT) program performed for 3 to 6 weeks is able to promote significant improvements in RE, PTS, and OBLA in trained participants (5,12–16). For instance, Smith et al. (15) applied a 4-week HIIT program to well-trained runners and observed a significant increase in PTS. In addition, Smith et al. (16) reported significant improvements in VO_2_max and RE in a group of well-trained runners after a 4-week HIIT. Based on these findings, one could hypothesize that the inclusion of a HIIT program can improve physiological variables related to endurance performance and, therefore, alter pacing strategy. However, to the best of our knowledge, no previous study has investigated the effects of a HIIT program on self-selected pacing during a 5-km running trial.

Therefore, the main purpose of this study was to analyze the influence of a 4-week HIIT program on pacing strategy during a 5-km running trial. Our hypothesis was that the HIIT program might improve physiological variables related to running pacing strategy (e.g., in RE, PTS, and OBLA), resulting in an altered U*-*shaped speed curve (i.e., a more intense and faster start).

## Material and Methods

### Participants

The sample size required was estimated using 5-km running performance as the main outcome from the equation n=8e^2^/d^2^, as proposed by Hopkins (17), where n, e, and d denote predicted sample size, coefficient of variation, and the magnitude of the treatment effect, respectively. The coefficient of variation was assumed to be 1.7% (18). Expecting a 2.8% magnitude of effect for the treatment (16), the detection of a very conservative 2% difference as statistically significant would require at least 5 participants for each group. However, to allow for any possible sample dropout, we targeted 8 participants per group. Thus, sixteen male long-distance runners were invited to participate in the present study. All participants were recreational runners from local clubs. The participants were included if they had participated in 5-km running races during the last two years, their best performance in the 5-km running races had been under 25 min, and if they had not participated in any HIIT program 6 months before the start of this study. They performed only low-intensity, continuous aerobic training (50–70% VO_2_max) before the beginning of the study and were instructed to maintain this aerobic training schedule during the experimental period. The participants’ running training volume was reported as the mean distance covered per week (19,20), which was assessed through a training log recorded for two weeks prior to the beginning of the study and for the last two weeks before the study completion. The participants were assigned to the HIIT group (n=8, age 35±6 years, body mass 70.5±4.6 kg, height 172.5±4.1 cm) or a control group (CON, n=8, age 32±9 years, body mass 70.2±11.3 kg, height 172.8±9.0 cm). The groups were matched for pre-training 5-km running overall performance. All of the participants were medication-free, non-smokers, and were free of neuromuscular disorders and cardiovascular dysfunctions. The participants received a verbal explanation about the possible benefits, risks, and discomfort associated with the study and signed a written informed consent before enrollment. The procedures adopted in this study were approved by the Ethics Committee for Human Studies from the School of Physical Education and Sport, University of São Paulo.

### Experimental design

Before and after the training intervention, the runners were required to visit the laboratory on three separate occasions, at least 72 h apart, over a 2-week period. During the first session, anthropometric measurements and a 5-km running trial on an outdoor track to establish pacing strategy were performed. The 5-km running trial was repeated 48 h after the first training session. The runners were familiar with long-distance running since they regularly competed in 5-km running events. During the second session, an incremental exercise test to exhaustion on a treadmill was conducted to determine the OBLA and VO_2_max. During the third session, the participants performed a submaximal constant-speed test on a treadmill to measure the RE. During the pre-training period, only the HIIT group performed a constant-speed running test at the speed corresponding to VO_2_max (vVO_2_max) to determine time to exhaustion at this speed (T_Lim_), which was used for individualizing the HIIT program (13). All tests were performed at the same period of day, and the first and second sessions were established randomly. All the participants were instructed to refrain from any exhaustive or unusual exercise 48 h before the test and to refrain from taking nutritional supplements during the training period. During training period, the HIIT program was added to the regular training schedule of HIIT group, while the participants of the CON group were instructed to maintain their regular training.

### Maximal incremental treadmill test

Participants performed a maximal incremental test on a motor-driven treadmill (model TK35, Cefise, Brazil). After a 3-min warm-up at 8 km/h, the speed was increased by 1 km/h every three minutes until exhaustion. The treadmill was set at a gradient of 1% to simulate physiological demand during outdoor running (21). Each stage was separated by a 30-s rest in which blood samples (25 µL) were collected from the ear lobe to determine blood lactate accumulation. The participants received strong verbal encouragement to ensure the attainment of maximal effort. Gas exchange was measured breath-by-breath using a gas analyzer (Cortex Metalyzer 3B, Cortex Biophysik, Germany) and subsequently averaged over 30-s intervals throughout the test. Before each test, the gas analyzer was calibrated according to the recommendations of the manufacturer. The VO_2_max was determined as the highest 20-s value reached during the last stage of the incremental test (13). The vVO_2_max was defined as the speed at which VO_2_max was achieved. The OBLA was defined as the running speed associated with 3.5 mmol/L of lactate concentration (22). PTS was established as the highest speed obtained in the last stage maintained for at least 3 min.

### Running economy

The RE was determined on a motor-driven treadmill (model TK35, Cefise). Participants performed a standardized warm-up, consisting of 5 min of running at 8 km/h followed by a 5-min passive recovery. Thereafter, they performed a constant-speed running test at 12 km/h for 10 min in order to measure the RE. During the entire test the oxygen uptake was obtained breath-by-breath. RE was defined by averaging the oxygen uptake values during the last 30 s.

### Time to exhaustion at the speed corresponding to VO_2_max

The participants in the HIIT group performed the same warm-up routine adopted during the RE test. The vVO_2_max was immediately adjusted after the warm-up and the participants ran until they could no longer maintain the required speed. The test began with the participant’s feet on the moving belt and hands on the handrail. The T_Lim_ was measured using a manual stopwatch and defined as the moment that the participant released the handrail (about 2 s) until he grasped it again (i.e., exhaustion). The participants received strong verbal encouragement to continue as long as possible.

### 5-km running trial

Participants individually performed a 5-km running trial on an outdoor 400-m track. They were instructed to maintain regular water consumption within the six hours prior to testing and water was provided *ad libitum* during the entire event. The runners performed a 10-min, warm-up consisting of a free-paced run, followed by 5 min of light stretching. The RPE was reported by participants every 1000 m using the Borg 15-point scale (23). Copies of this scale were reduced to 10 by 5 cm and laminated, and affixed to the wrist of the dominant arm of the individuals. The participants were instructed to finish the race as quickly as possible, as in a competitive event. Verbal encouragement was provided during the entire event. However, runners were not advised of their lap splits. Time to cover the 5-km (T5) and heart rate (HR_T5_) were registered by a GPS every 400 m (GPS Forerunner® 410, USA). The pattern of data collecting for RPE (at 1000 m intervals), T5, and HR_T5_ (both at 400 m intervals) was according with a previous study carried out by Lima-Silva et al. (9). All tests were performed at the same time of the day and the mean values of the ambient temperature and air relative humidity were 19±4°C and 59±5%, respectively.

### Training program

The HIIT group performed a high-intensity interval training program twice weekly (separated by 48 h) for 4 weeks in addition to their normal endurance training. The athletes were instructed to perform their regular endurance training on different days to those of the HIIT sessions. In order to equal the training load between the training regimes, there was a reduction of ∼10% of the total endurance training volume (i.e., km/week) in the HIIT group. A standardized warm-up consisting of a 5-min run at 9 km/h followed by light lower-limb stretching exercises was performed before each training session. Because T_Lim_ is assumed to be a useful tool for intermittent training prescription (13), in the present study athletes completed five intervals at the vVO_2_max for a duration equal to 50% of the T_Lim_, interspersed with an active recovery at 60% of the speed corresponding to vVO_2_max for a duration equal to the time of effort (i.e., 1:1 work:recovery ratio). The running speed during the HIIT was monitored by a GPS (GPS Forerunner¯ 305). The training sessions were individually supervised to control the training loads. Over this 4-week training period, the CON group was instructed to maintain their previous endurance training routine.

### Statistical analysis

Data normality was confirmed using the Shapiro-Wilk test. Two-way analysis of variance (group × time) was used to compare the physiological and performance variables. In order to mitigate the impact of inter-individual data variability, physiological variables are also reported as percentage of change from pre-training period (i.e., Post-Pre). Comparison between groups for percent of changes (%) after the experimental period was performed using unpaired *t*-test. Significance was accepted at P<0.05. All statistical analyses were performed using the software package Statistica 8 (StataSoft Inc., USA).

## Results

### Training

All of the participants in the HIIT group completed over 85% of the scheduled training sessions. The mean value of T_Lim_ used for prescription of HIIT was 265±67 s. No statistical difference was observed in the endurance training volume (reported as the mean weekly covered distance) between before (HIIT: 28.7±2.3 km/week, CON: 30.2±1.3 km/week) and after (HIIT: 29.3±9.8 km/week, CON: 32.1±2.3 km/week) the completion of the study (P*>*0.05), indicating that training load was equal between training regimes.

### Physiological variables

**Figure 1.**
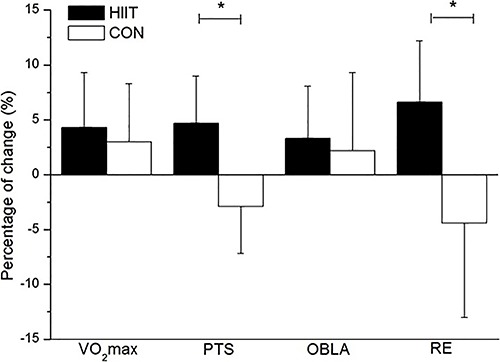
Percentage of changes of the physiological variables after the training period. Data are reported as means±SD. HIIT: high-intensity interval training group; CON: control group; VO_2_max: maximal oxygen uptake; PTS: peak treadmill speed; OBLA: running speed associated with onset of blood lactate accumulation; RE: running economy measured at 12 km/h. *P<0.05 (unpaired *t*-test).

Figure 1 shows the relative changes in the physiological parameters measured during the maximal incremental and constant-speed running. After the experimental period, the HIIT program produced significant improvements in RE (P*=*0.012) and PTS (P*=*0.019) when compared with the CON group. There was no significant difference between the groups for VO_2_max (P*=*0.495) and OBLA (P*=*0.101). Table 1 presents the absolute values of the physiological parameters measured during the maximal incremental and constant-speed running. There were no main effects for time (P*>*0.05), group (P*>*0.05), or interaction (P*>*0.05) for all measured variables.

**Table 1.** Parameters related to endurance performance before and after the 4-week high-intensity interval training period.

	HIIT (n=8)		CON (n=8)	
	Pre	Post	Pre	Post
V̇O_2_max (mL·kg^-1^·min^-1^)	54.5±8.1	57.1±6.4	56.6±7.3	56.9±7.6
PTS (km/h)	16.5±1.8	17.2±1.8	17.9±1.0	17.7±1.6
OBLA (km/h)	14.1±2.3	15.0±2.4	15.1±2.2	15.3±1.8
RE (mL·kg^-1^·min^-1^)	43.1±3.5	40.7±4.3	40.9±4.7	41.2±4.4

Data are reported as means±SD. HIIT: high-intensity interval training group; CON: control group; V̇O_2_max: maximal oxygen uptake; PTS: peak treadmill speed; OBLA: running speed corresponding to onset of blood lactate accumulation; RE: running economy.

### 5-km running trial

**Figure 2.**
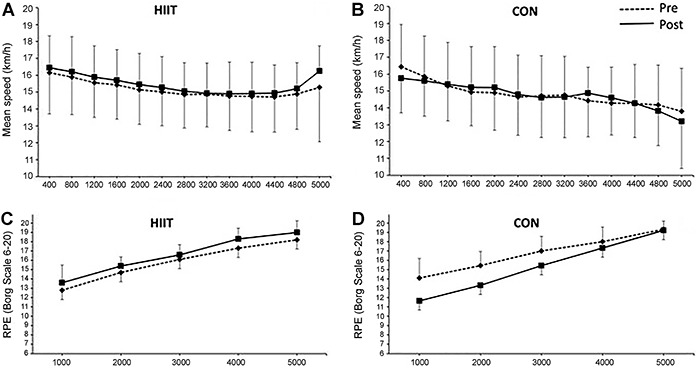
Running pacing strategy (*panels A* and *B*) and rating of perceived exertion (RPE; *panels C* and *D*) during a 5-km running trial, pre- and post-training. Data are reported as means±SD. HIIT: high-intensity interval training group; CON: control group.

Table 2 presents the main variables measured during the 5-km running trial. All parameters measured during the 5-km trial before the training period were the same between HIIT and CON groups (P*>*0.05). There were no significant main effects for time, group, nor interaction effects for T5, HR_T5_, and RPE_T5_ (P*>*0.05). Figure 2 shows the pacing strategy and RPE during the time trial before and after training. No significant main effect was observed for either variable (P*>*0.05).

**Table 2.** Running performance, heart rate, and rate of perceived exertion during a 5-km running trial pre- and post-training.

	HIIT		CON	
	Pre	Post	Pre	Post
T5 (s)	1196±173	1168±135	1149±153	1165±164
HR_T5_ (bpm)	178±4	176±5	174±8	172±9
RPE_T5_ (score)	17±1	17±2	17±1	16±1

Data are reported as means±SD. HIIT: high-intensity interval training group; CON: control group; T5: time to cover; HR_T5_: mean heart rate at T5; RPE_T5_: mean rate of perceived exertion during the 5-km running trial.

## Discussion

The main objective of the present study was to investigate the effects of the addition of a 4-week HIIT program on the pacing strategy adopted by long-distance runners during a 5-km running trial. The main findings were that the HIIT program improved physiological variables related to endurance performance (i.e., RE and PTS), but these changes were not accompanied by modifications in pacing strategy or overall performance.

Previous findings have showed that a similar HIIT protocol was able to improve some physiological variables related to endurance performance (13). Although there was no significant difference between the groups for the absolute values of the physiological variables after the training period, our findings revealed that the addition of 4-week HIIT program produced significant improvements in percentage of change in PTS and RE, corresponding to a mean improvement of 5.6 and 4.1%, respectively. These data are in agreement with several studies that have reported similar improvements of ∼4.4% in the vVO_2_max (13,15) and ∼5% in the RE (13,14,16) after a 4-week HIIT program. Specifically, it has been proposed that PTS is influenced not only by maximal aerobic power, but also by RE (24,25). An improvement in RE after the HIIT program could lead to a lower energy cost during submaximal running bouts, which might allow the athletes to achieve higher speeds at the end of the maximal incremental treadmill test. Therefore, it seems that the main beneficial effects of the HIIT program are mediated by a reduction in the energetic cost of running. Taken together, these findings reinforce the suggestion that a HIIT program performed during 4 weeks is an effective short-term strategy to alter some physiological variables related to endurance performance.

In the present study, we have provided the first data analyzing the effectiveness of a HIIT program on the pacing strategy adopted by endurance runners during a long-distance event. It was found that although the percentage of changes of the PTS and RE were improved after the HIIT program, the pacing strategy was maintained after the experimental intervention. Previous studies have proposed that pacing strategy can be controlled by a centrally-regulated system that monitors the RPE in order to minimize physiological strain and to prevent a premature exercise termination (26,27). It has been proposed that the CNS interprets afferent feedback from physiological systems in order to adjust the work performed by skeletal muscles and avoid premature fatigue (28). Thus, the RPE is the integration of alterations in physiological systems used during dynamic exercise and is considered a primary regulator of pacing strategy (27). This has led some researchers to hypothesize that interventions (i.e., physical training and dietary manipulation) that change these physiological variables could influence the RPE, resulting in an altered pacing strategy (28). However, the data of the present study revealed that the HIIT program improved the RE and PTS (Figure 1), but without changes in RPE (Figure 2). These findings suggest that improvements in physiological variables would produce only a small reduction in metabolic disturbance during exercise of self-paced intensity. This could result in a similar afferent feedback from physiological systems when compared with pre-training. Thus, the interpretation of the afferent feedback during running was not altered after the HIIT program, as revealed by RPE, and athletes adopted a similar pacing strategy to that used before the training period. These findings are in agreement with a previous suggestion that athletes adjust their pacing strategy by comparing actual and expected RPE during the course of a race for a given distance (29).

The improvement of only ∼2.5% in 5-km running performance detected in the present study is in agreement with others that reported small enhancement in overall running performance after a 4-week HIIT program (13,15,16). For instance, Smith et al. (15) found a 2.7% improvement on 3000-m running performance after a HIIT program, while both VO_2_max and vVO_2_max showed a significant increase (∼4.9%). Smith et al. (16) verified that a similar HIIT program was able to promote improvements of around 6.0% in VO_2_max, 5.2% in vVO_2_max, and a non-significant improvement in 5-km running performance. In addition, Billat et al. (13) found non-significant changes in 3000-m running performance after a 4-week HIIT program. Taken together, these findings suggest that the improvements in physiological variables (i.e., 3–6%) produced by a short-term HIIT program were not translated to improved endurance performance. The reasons for this stable running endurance performance after HIIT programs are not clear, but it is possible that moderate improvements on physiological variables are not enough to reduce afferent feedback from physiological systems when compared with pre-training. This could explain the non-significant change in perception of effort found in the present study, producing only a small improvement in overall running performance.

It is important to acknowledge some of the limitations of the present study. First, our participants were recreational long-distance runners who had only low-intensity continuous aerobic training experience. Thus, caution should be taken in extrapolating these findings to highly-trained athletes who frequently perform HIIT training sessions. Second, the athletes individually performed the 5-km running trial, while during official competitive running races, they compete in a head-to-head manner. Previous findings have suggested that the presence of other competitors would alter the pacing strategy, inducing to a more aggressive and faster start and improving overall performance (2). This could limit the extrapolation of the findings of the current study to a more realistic scenario of endurance competition. Thus, future studies are encouraged to verify the impact of the HIIT on running pacing strategy determined in a head-to-head manner.

In conclusion, the results of the present study showed that the addition of 4 weeks of HIIT produced relevant gains on the PTS and RE, but without changes in RPE, pacing strategy, and overall performance. These findings suggest that improved aerobic power and lower energy cost during submaximal running were not sufficient to alter the perceived effort behavior during a 5-km running trial, resulting in a similar pacing strategy to that used before the training period.
